# Modeling for Predicting the Time to Detection of Staphylococcal Enterotoxin A in Cooked Chicken Product

**DOI:** 10.3389/fmicb.2018.01536

**Published:** 2018-07-13

**Authors:** Jieyun Hu, Lu Lin, Min Chen, Weiling Yan

**Affiliations:** ^1^Shanghai Food Research Institute, Shanghai, China; ^2^Shanghai Municipal Center for Disease Control and Prevention, Shanghai, China

**Keywords:** predictive microbiology, *Staphylococcus aureus*, Staphylococcal enterotoxins, time to detection, cooked chicken

## Abstract

Staphylococcal enterotoxins (SEs) produced by *Staphylococcus aureus* (*S. aureus*) are the cause of Saphylococcal food poisoning (SFP) outbreaks. Thus, estimation of the time to detection (TTD) of SEs, that is, the time required to reach the SEs detection limit, is essential for food preservation and quantitative risk assessment. This study was conducted to explore an appropriate method to predict the TTD of SEs in cooked chicken product under variable environmental conditions. An *S. aureus* strain that produces staphylococcal enterotoxin A (SEA) was inoculated into cooked chicken meat. Initial inoculating concentrations (approximately 10^2^, 10^3^, 10^4^ CFU/g) of *S. aureus* and incubation temperatures (15 ± 1, 22 ± 1, 29 ± 1, and 36 ± 1°C) were chosen as environmental variables. The counting of *S. aureus* colonies and the detection of SEA were performed every 3 or 6 h during the incubation. The TTD of SEA was considered a response of *S. aureus* to environmental variables. Linear polynomial regression was used to model the effects of environmental variables on the TTD of SEA. Result showed that the correlation coefficient (*R*^2^) of the regressed equation is higher than 0.98, which means the obtained equation was reliable. Moreover, the minimum concentration of *S. aureus* for producing a detectable amount of SEA under various environmental conditions was approximately 6.32 log CFU/g, which was considered the threshold for *S. aureus* to produce SEA. Hence, the TTD of SEA could be obtained by calculating the time required to reach the threshold by using an established *S. aureus* growth predictive model. Both established methods were validated through internal and external validation. The results of graphical comparison, *RMSE, SEP, A*_*f*_, and *B*_*f*_ showed that the accuracy of both methods were acceptable, and linear polynomial regression method showed more accurately.

## Introduction

*Staphylococcus aureus* (*S. aureus*) is a worldwide cause of foodborne diseases. In the European Union (EU), 393 foodborne outbreaks caused by staphylococcal species were reported in 2014 (EFSA, 2014). In the US, foodborne illnesses caused by *S. aureus* were estimated to range from 72,341 to 529,417 (Scallan et al., 2011). According to the Notification of National Food Poison Outbreaks in 2015 issued by the National Health Commission of P. R. China (http://www.nhfpc.gov.cn/yjb/s7859/201604/8d34e4c442c54d33909319954c43311c.shtml), *S. aureus* is one of the major causes of foodborne diseases in China.

Ready-to-Eat (RTE) cooked meat products are very popular in China, the poultry is the major ingredient of RTE cooked meat products. In many local delis, RTE meat products are stored at room temperature, and almost all the products are stored without packaging or with very simple packaging (wrapped with polyethylene film) to prevent dust. In addition, some of these products such as cooked chickens or ducks would be sliced and displayed in counter for a quite long time before sell. This made them susceptible to contamination by microorganism including pathogens from the environment during the storage (Denayer et al., 2017). Several food safety monitoring studies in China (Ye et al., 2012; Chen et al., 2014; Zhou et al., 2014) indicated that *S. aureus* was one of the most common foodborne pathogens associated with cooked meat products.

Staphylococcal enterotoxins (SEs) are synthesized by *S. aureus* in food if growth conditions are adequate for the microorganism (Smith et al., 1983). It is a family consists of more than 20 serologically different SEs, share a sequence homology (Sospedra et al., 2013). SEs are capable of causing gastroenteritis in humans, thereby making them the causative agents of SFP (Wu and Su, 2014). In addition, SEs are resistant to proteolytic enzymes and normal heat processing; even if *S. aureus* have been sterilized, the biological activity of SEs remains unchanged. According to regulation (EC) No 1441/2007, if the coagulase-positive staphylococci (CPS, mainly *S. aureus*) counts are >10^5^ CFU/g during the processing, then the testing of enterotoxins must be performed. Given that the SEs and not *S. aureus* itself caused the SFP, and the detection of SEs was reportedly an in-depth process (Cretenet et al., 2011), a question was raised: supposing the foodstuff was contaminated by *S. aureus*, is estimating the time to detection (TTD) of SEs (the time required to reach the SEs detection limit) in food possible? If so, how can this be done? Numerous studies on *S. aureus* growth or survive predictive models in meat (Rodriguez-Caturla et al., 2009; Valero et al., 2009; Min et al., 2012; Lee et al., 2015) had been developed. However, less attention was paid to predictive staphylococcal enterotoxin models, which are essential for ruling regulation, food preservation, and quantitative risk assessment (Schelin et al., 2010).

Similar to the survival and growth of *S. aureus*, which are environment dependent, SE production in different foods during processing and storage was also considered environment dependent in many articles (Smith et al., 1983; Wallin-Carlquist et al., 2010; Tsutsuura et al., 2013). Therefore, in the present study, we assumed that the TTD of SEs in food was a response of *S. aureus* to environmental variables. The effects of environmental variables on the growth kinetics (such as lag phase and maximal rate) of bacteria could be established by the construction of a second model. Thus, we assumed that the SEs could also be predicted by establishing a mathematical model that describes the effects of environmental variables on the TTD of SEs.

In this study, we attempted to use linear polynomial regression to quantify the effect of environmental variables on the TTD of SEs in food. Considering that the validity of applying predictive models constructed in the laboratories in real food matrix is often questioned, an *S. aureus* strain that produces enterotoxin A (SEA), the most common type of SEs in food products (Smith et al., 1983; Tsutsuura et al., 2013; Zeaki et al., 2014), was chosen for inoculation into cooked chicken meat. The *S. aureus* colonies were counted and the SEA was detected regularly during the incubation. Linear polynomial regression was applied to model the effects of environmental variables the TTD of SEA.

## Materials and methods

### Experimental design

To understand the basic technological profile of RTE cooked meat products, more than 20 cooked meat products were purchased from several delis around Shanghai. The NaCl level of these samples was tested in accordance with the China National Standards (GB/T12457-2008). Water activity (a_w_) and pH value were tested by an Aw meter (HygroLab2, Rotronic,) and a pH meter (Delta 320, Mettler Toledo, China) in accordance with GB 5009.238-2016 and GB 5009.237-2016, respectively. Results showed that the NaCl level, water activity (a_w_), and pH value of all the samples ranged from 0.5–2.2%, 0.960–0.980, and 6.52–6.84, respectively. *S. aureus* is well known for its tolerance to salt, low a_w_, and acidic environment. A previous study (Smith et al., 1983) revealed that the NaCl level required for *S. aureus* to produce enterotoxins ranged from 0 to 10%, and initial pH values of 5.0–8.0 yielded similar amounts of SEA production.

The above finding indicates that the NaCl level and pH value of RTE cooked chicken product in China appear to be unlikely to significantly affect the growth rate and SEs production of *S aureus*. Therefore, in this study, cooked chicken was selected as the food matrix, and various incubation temperatures and initial inoculating concentrations were chosen as the critical variables to develop the model (Table 1). Six additional experiment conditionals were used to validate the predicting model (Table 2).

**Table 1:** Environmental factors and experimental design for modeling the TTD of SEA in cooked chicken product.

**Incubation temperature (°C)**	**Initial inoculation level of *S. aureus* (CFU/g)**	**Sampling interval (h)**	**Duration of experiment (h)**
15 ± 1	10^2^, 10^3^, 10^4^	6	72
22 ± 1	10^2^, 10^3^, 10^4^	6	72
29 ± 1	10^2^, 10^3^, 10^4^	3	36
36 ± 1	10^2^, 10^3^, 10^4^	3	36

**Table 2:** Additional experiment groups designed for validating the predicted model.

**Incubation temperature(°C)**	**Initial inoculation level of *S. aureus* (CFU/g)**	**Sampling interval (h)**	**Duration of experiment (h)**
25 ± 1	10^2^, 10^3^, 10^4^	6	72
32 ± 1	10^2^, 10^3^, 10^4^	6	72

#### Fresh chicken meat

A 500 g packet of chilled skinless chicken breasts (Tyson Food, Inc. China) was purchased at a local supermarket. The chilled skinless chicken breasts was determined to be *S. aureus*—free when samples were tested by a standard spread plate count assay (China National Food Safety Standards GB 4789.10-2010) and was verified to be SEA - free based on the results of the VIDAS SET2 described below.

#### *S. aureus* strain and preparation of inoculum

An *S. aureus* strain (SA14966) that is known to produce SEA was isolated from food and preserved in Shanghai Food Research Institute (Wang, 2015). To prepare the inoculum, 0.1 mL of thawed bacteria was moved into 50 mL of Luria–Bertani (LB) medium and were grown to the cell concentration of approximately 9.0 log Colony-Forming Units (CFU)/mL in a thermostatic oscillating incubator (THZ-100, YiHeng, Shanghai, China) at 100 rpm at 36 ± 1°C for 18–20 h. Single colonies of bacteria were obtained from plate streaking, which were incubated at 36 ± 1°C for 24 h. Referencing the method of Peter and Robert (2005), bacteria obtained from a single colony was transferred into 50 mL of LB medium, and incubated in a thermostatic oscillating incubator at 100 rpm at 36 ± 1°C for 18–20 h. Then, the bacteria were centrifuged using a refrigerated benchtop centrifuge(H-2050R, XiangYi, HuNan, China) at 12,000 rpm at 4°C for 30 min and washed with sterile 0.1 mol/L potassium phosphate buffer (pH 7) solution two times and diluted to the desired concentration. Two times' washing guarantees that the resulting solution contains only *S. aureus* cells without carrying SEA.

#### Sample preparation

Fresh chicken meats were divided into blocks and cooked in boiling water for 10 min. After cooling and draining, the cooked chicken meat was then immersed into an *S. aureus* solution with various cell concentrations for approximately 5 min. According to the experimental design of this study, the initial inoculation concentration of sample was designed to 10^2^, 10^3^, and 10^4^ CFU/g separately. To guarantee the initial inoculation concentration of the sample close to the designed concentration, the desired concentration of *S. aureus* solution for inoculating was diluted to 10-times higher than the designed concentration; the sample to volume of suspension ratio was set to 1:5. The actual initial inoculation concentration of the sample would be confirmed based on the method described below.

Each 50 g inoculated chicken meat was then packaged into a sterile plastic bag under a clean bench and stored at 15 ± 1, 22 ± 1, 29 ± 1, and 36 ± 1°C. The samples were tested every 3 or 6 h to count the numbers of colonies.

#### Plate counting of *S. aureus* and detetion of staphylococcal enterotoxin A

About 25 g chicken meat from each sample was homogenized with 225 mL of sterile saline (0.9%) following shaking for 2 min with a stomacher (Analis BagMixer 400 p, Interscience, France). Viable counts were carried out by plating a dilution in sterile saline onto 3 M Petrifilm™ Staph Express Count Plate (3M Center, St. Paul, MN 55144,USA) after being incubated at 36 ± 1°C for 24 h. A total of 25–30 g residual from each sample was used to detect the SEA.

The VIDAS Staphylococcal Enterotoxin II (SET2) detection kits (Biomerieux, France) and a Mini-VIDAS auto analyzer (Biomerieux, France) were applied for the detection of SEA in this study. The principle of this measurement system is enzyme-linked fluorescent assay using polyclonal anti-enterotoxin antibody. Results are expressed as TV, which is the relative fluorescence value (RFV) of the test solution divided by the RFV of the standard in the VIDAS SET2 kit. A test solution with a TV value of ≥0.13 was considered positive. The detection limit is 0.5 ng/g of SEA.

Three replicates of plate counting and SEA detection were performed for each condition. The average values and the standard deviations of the transformed values were then calculated.

### Correction of the observed TTD of SEA and the estimation of *S. aureus* concentration at the TTD of SEA

**Figure 1:**
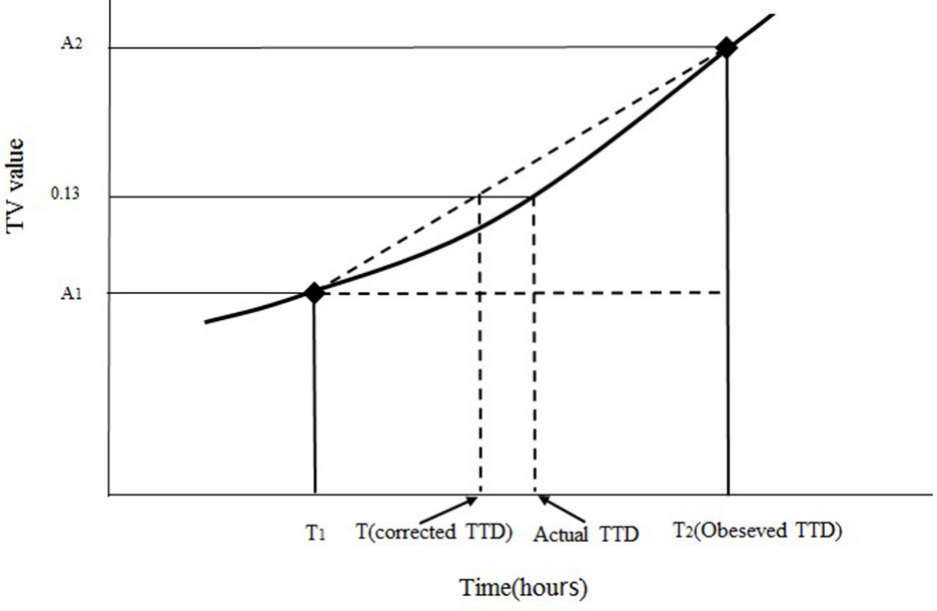
Diagram of the correction for obtaining the corrected TTD of the SEA. *A*_1_ is the TV value of the last negative point at the time of *T*_1_, *A*_2_ is the TV value of the first positive point at the time of *T*_2_, *T* is the corrected TTD of SEA. The curve with (♦) is the trend of TV value during incubation.

One of the major factors that influence the successful establishment of the model is the determination of the actual TTD of SEA under designed conditions. Given the 3 or 6 h sampling interval in this study, the observed TTD of SEA (the first positive point) will not be the same as the actual TTD of SEA (TV = 0.13). To obtain the time close to the actual TTD of SEA, linear interpolation was used to correct the observed TTD. The linear interpolation approach is shown in Figure 1. The corrected TTD of SEA was close to the actual TTD of SEA.

In accordance with Figure 1 and the principle of similar triangles, the corrected TTD of SEA could be obtained from Equation (1) (Tu and Huang, 2012).

(1) $$\begin{array}{l} T=T_{1}+\frac{\left(T_{2}-T_{1}\right)\left(0.13-A_{1}\right)}{A_{2}-A_{1}} \end{array}$$

where *T* is the corrected TTD of SEA, *A*_1_ is the TV value of the last negative point at the time of *T*_1_, *A*_2_ is the TV value of the first positive point at the time of *T*_2_, and 0.13 is the TV value at the SEA detection limit.

The concentration of *S. aureus* at the observed TTD of SEA was obtained by *S. aureus* counting from that point. The concentration of *S. aureus* at the corrected TTD of SEA could be obtained by substituting *T* into the modified Gompertz equation under the studied temperature.

### Construction and validation of the model

#### Modeling of primary model for *S. aureus* growth

According to the results of our previous study (Hu et al., 2016), the modified Gompertz equation (Gibson et al., 1987) was appropriate to describe the *S. aureus* growth in cooked chicken meat under the temperature range of 15 ± 1–36 ± 1°C.

#### Modeling of linear polynomial regression

In this study, various initial inoculation concentrations and incubation temperature were chosen as the critical variables, and linear polynomial regression was used to model the effect of variables on the TTD of SEA. MATLAB2014a was used to regress the model. The linear polynomial regression was established as follows (Zhao et al., 2002; Basti and Razavilar, 2004):

(2) $$\begin{array}{l} TTD=a_{0}+a_{1}Linoc+a_{2}T+a_{3}T^{*}Linoc+a_{4}Linoc^{2}+a_{5}T^{2} \end{array}$$

where *Linoc* is the log(initial inoculation concentration) (CFU/g), *T* is the incubation temperature (°C), and *a*_i_ is the coefficient estimated by regression (i = 0,1,2,… 5). The corrected TTD of SEA was used as the observed value during regression.

#### Validation and reliability evaluation of the model

After the establishment of a predictive model, predicted values from 12 conditions for model establishment (Table 1) and additional 6 conditions (Table 2) for model validation were mathematically evaluated. The corrected TTD of SEA from all the conditions were regarded as observed values. In this paper, the accuracy of the predictive model describing the TTD of SEA was evaluated by four criteria: root mean-square error *(RMSE)*, standard error of prediction *(SEP)* (Hervas et al., 2001; Garcia-Gimeno et al., 2005; Zurera-Cosano et al., 2006), bias factor *(B*_*f*_*)*, and accuracy factor *(A*_*f*_*)* (Ross, 1996; Garcia-Gimeno et al., 2005; Zurera-Cosano et al., 2006), which are shown as follows:

(3) $$\begin{array}{l} B_{f}=10^{\left(\frac{\sum \log\left(pred/obs\right)}{n}\right)} \end{array}$$

(4) $$\begin{array}{l} A_{f}=10^{\left(\frac{\sum |\log\left(pred/obs\right)|}{n}\right)} \end{array}$$

(5) $$\begin{array}{l} \%SEP=\frac{100}{meanobs}\sqrt{\frac{\sum {\left(obs-pred\right)}^{2}}{n}} \end{array}$$

(6) $$\begin{array}{l} RMSE=\sqrt{\frac{\sum {\left(obs-pred\right)}^{2}}{n}} \end{array}$$

where *obs* is the observed value, *pred* is the predicted *TTD* calculated from the constructed predictive method, and meanobs is the mean of the observed value.

### Statistical analysis

An analysis of variance (ANOVA) of the established equation was performed and the correlation test of the variables on the TTD of SEA was measured by using SPSS Statistics 23.0.

## Results

### Predictive model of TTD of SEA using linear polynomial regression

**Figure 2:**
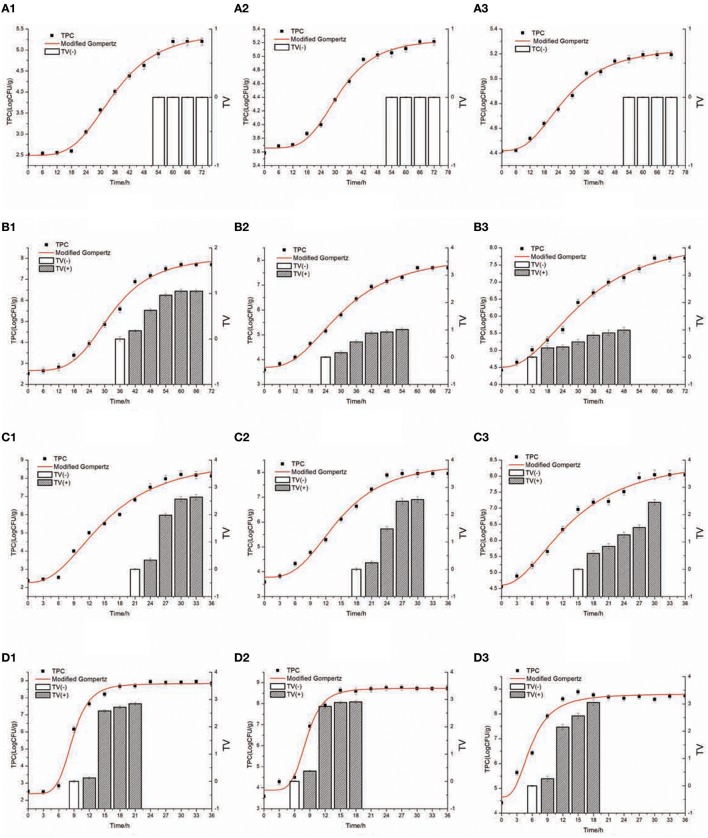
*S. aureus* growth and SEA production in cooked chicken meat at 15–36°C [**(A)** 15°C, **(B)** 22°C, **(C)** 29°C, **(D)** 36°C] with inoculating concentration approximately (1) 10^2^CFU/g, (2) 10^3^ CFU/g, (3) 10^4^CFU/g. TV value (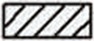) of ≥0.13 was considered positive and SEA produced; TV value (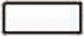) of ≤0.13 was considered negative and no SEA produced. The curved line with (♦) shows measured viable cell counts, and the curved line without squares shows the curve modeled by the modified Gompertz model.

Figure 2 presents the *S. aureus* growth and SEA production in cooked chicken meat under 15 ± 1, 22 ± 1, 29 ± 1, and 36 ± 1°C with Initial inoculating concentrations approximately 10^2^, 10^3^, and 10^4^ CFU/g. Table 3 shows the observed TTD of SEA, corrected TTD of SEA, and the concentration of *S. aureus* at the corrected TTD of SEA under various temperatures and initial inoculation concentrations. The TTD of SEA at 15°C could not be found in Table 3, because the SEA could not be detected at 15°C within 72 h. Thus, the temperature range for model regression was set from 22 to 36°C.

**Table 3:** TTD of SEA in cooked chicken meat under various combinations of incubation temperatures and initial inoculation concentrations of *S. aureus*.

**Incubation temperature(°C)**	**Designed initial inoculation concentration (log CFU/g)**	**Actual initial inoculation concentration (SD) (log CFU/g)**	**Maximum concentration of *S. aureus* during incubation (SD) (log CFU/g)**	**Observed TTD (h)**	**Concentration of *S. aureus* at the observed TTD (SD) (log CFU/g)**	**Corrected TTD (SD) (h)**	**Concentration of *S. aureus* at the corrected TTD (SD) (logCFU/g)**
15 ± 1	2	2.51 (0.01)	5.19 (0.05)	nd	–	–	–
15 ± 1	3	3.59 (0.04)	5.21 (0.04)	nd	–	–	–
15 ± 1	4	4.42 (0.02)	5.21 (0.01)	nd	–	–	–
22 ± 1	2	2.51 (0.01)	7.69 (0.06)	42.0	6.88 (0.06)	36.55 (0.18)	6.03 (0.17)
22 ± 1	3	3.59 (0.04)	7.77 (0.02)	30.0	6.83 (0.08)	24.94 (0.15)	6.25 (0.14)
22 ± 1	4	4.42 (0.02)	7.83 (0.08)	18.0	6.40 (0.04)	17.20 (0.19)	6.28 (0.21)
29 ± 1	2	2.51 (0.01)	7.95 (0.09)	24.0	6.61 (0.02)	21.58 (0.13)	6.18 (0.16)
29 ± 1	3	3.59 (0.04)	8.03 (0.01)	21.0	7.31 (0.05)	18.41 (0.15)	6.75 (0.11)
29 ± 1	4	4.42 (0.02)	8.12 (0.01)	18.0	7.18 (0.05)	15.02 (0.11)	6.96 (0.09)
36 ± 1	2	2.51 (0.01)	8.72 (0.03)	12.0	7.64 (0.07)	9.05 (0.07)	6.17 (0.05)
36 ± 1	3	3.59 (0.04)	8.84 (0.02)	9.0	6.92 (0.06)	7.03 (0.05)	5.82 (0.03)
36 ± 1	4	4.42 (0.02)	8.84 (0.05)	9.0	7.91 (0.01)	6.04 (0.09)	6.43 (0.06)

*nd, no SEA was detected; –, no result*.

A linear polynomial regression model that describes the effects of temperature and initial inoculation concentration on the TTD of SEA was established by stepwise regression as follows:

(7) $$\begin{array}{l} TTD=112.9-23.95Linoc-1.677T+0.6122T\times Linoc\\ +0.1663{\left(Linoc\right)}^{2}-0.03133{\left(T\right)}^{2}\left({\text{R}}^{2}=0.9895\right) \end{array}$$

The ANOVA of the linear polynomial regression model indicated that Equation (7) was significant (*p* < 0.01). Thus, the regressed equation was reliable. On the basis of this finding, Equation (7) could be used to estimate the TTD of SEA under a temperature range of 22–36°C and an initial inoculation concentration range of 10^2^-10^4^ CFU/g. Therefore, this model was called the predictive model of TTD of SEA using linear polynomial regression.

On the basis of the linear polynomial regression equation of the obtained TTD predictive model (Equation 7), the effects of environmental variables (temperature and initial concentration) on the parameters (i.e., TTD of SEA) were analyzed. Even under the maximum initial inoculation concentration and temperature, the calculated value of the primary terms was much larger than that of the interaction term and quadratic terms. This condition means that the changes in values of TTD were mainly dependent on the primary terms of Equation (7). Given the negative coefficients of the primary terms, we can conclude that temperature and initial inoculation concentration have a decreasing effect on the values of the TTD. This conclusion is consistent with the observations of the experiments. For example, SEA can be detected 6–9 h after inoculation at 36°C. When the temperature decreased to 22°C, the TTD of SEA was delayed to 17–37 h. SEA could not be detected during 72 h at 15°C.

The result of correlation test for both independent factors also confirmed the effects of temperature and initial concentration on the TTD of SEA. The Pearson correlation coefficient (PCC) was −0.81, which meant the temperature was highly negative correlated with the TTD. The initial inoculation level also showed negative correlation with the TTD, but its correlation level was intermediate (PCC = −0.48).

**Figure 3:**
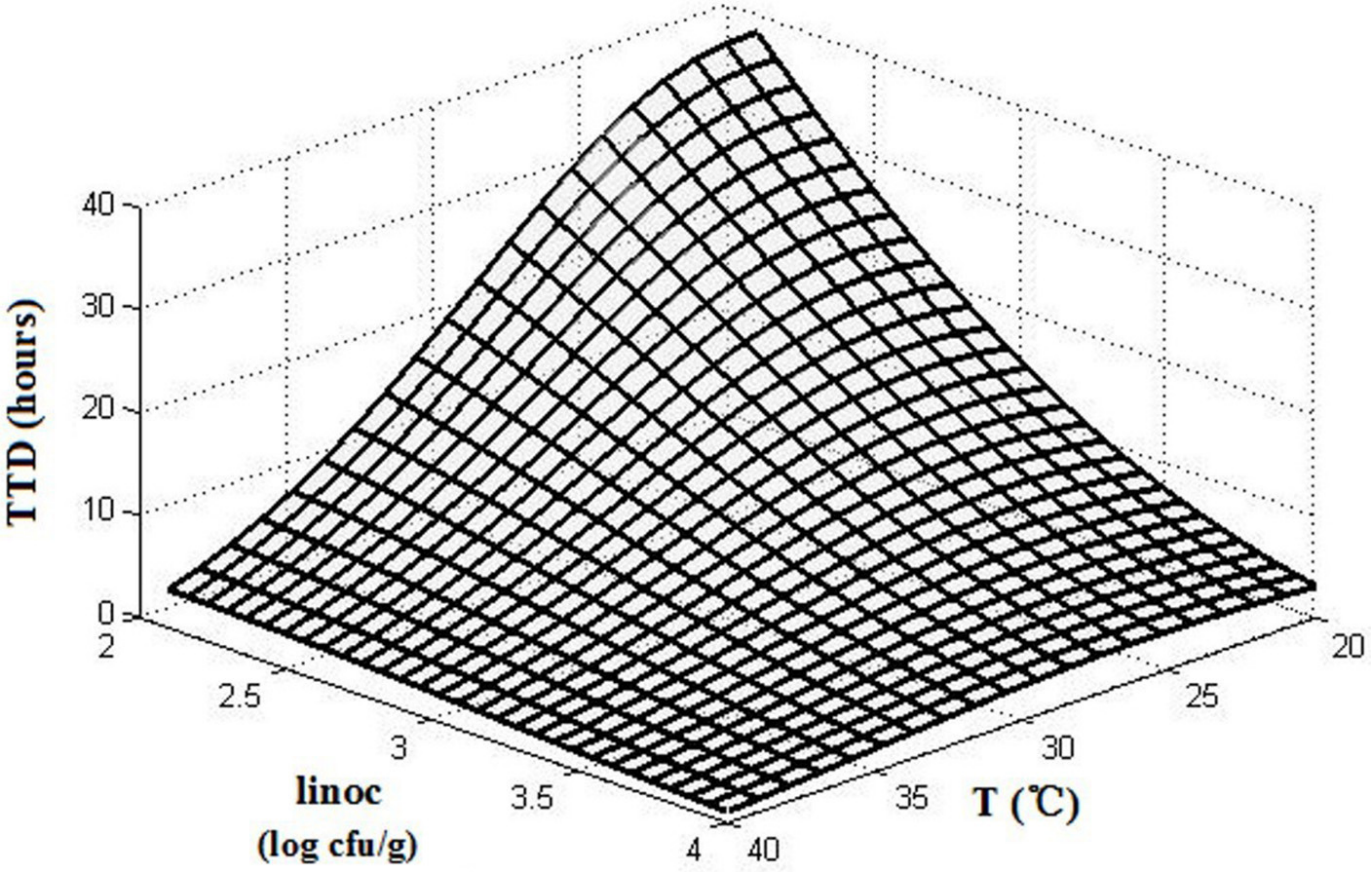
The 3D surface plots of TTD affected by temperature (T) and initial inoculation level (linoc).

The interactions of the environmental variables on the TTD of SEA are shown in a 3D surface plot (Figure 3). Figure 2 shows that the TTD of SEA decreased within the range of experimental limits with an increase in the temperature and initial inoculation level. Although both temperature and initial inoculation concentration had a negative effect on the TTD of SEA, the slopes of the combined plots of temperature and initial inoculation concentration indicated that the effect of incubation temperature was more significant (the direction of temperature is much steeper).

The predicted results of 12 combinations for model establishment and 6 combinations for model validation as obtained by this model are shown in Tables 4, 5, respectively.

**Table 4:** Observed and predicted TTD of SEA under combined conditions for internal validation.

**T (°C)**	**Inoculation level (SD) (log CFU/g)**	***Obs* (corrected TTD) (SD) (h)**	***Pred*** **(h)**	
			**Linear polynomial regression**	**Threshold method**
15 ± 1	2.51 (0.01)	nd	44.68	uc
15 ± 1	3.59 (0.04)	nd	29.83	uc
15 ± 1	4.42 (0.02)	nd	18.67	uc
22 ± 1	2.51 (0.01)	36.55 (0.18)	35.58	38.60
22 ± 1	3.59 (0.04)	24.94 (0.15)	25.35	26.53
22 ± 1	4.42 (0.02)	17.20 (0.19)	17.76	19.24
29 ± 1	2.51 (0.01)	21.58 (0.13)	23.42	19.63
29 ± 1	3.59 (0.04)	18.41 (0.15)	17.82	16.58
29 ± 1	4.42 (0.02)	15.02 (0.11)	13.78	13.24
36 ± 1	2.51 (0.01)	9.05 (0.07)	8.18	9.56
36 ± 1	3.59 (0.04)	7.03 (0.05)	7.21	8.36
36 ± 1	4.42 (0.02)	6.04 (0.09)	6.73	5.02

*nd, no SEA was detected; uc, uncalculated using the threshold method*.

**Table 5:** Observed and predicted TTD of SEA under combined conditions for external validation.

**T (°C)**	**Inoculation level (SD) (log CFU/g)**	***Obs* (corrected TTD) (SD) (h)**	***Pred*** **(h)**	
			**Linear polynomial regression**	**Threshold method**
25 ± 1	2.51 (0.01)	29.00 (0.12)	30.74	32.07
25 ± 1	3.59 (0.04)	23.00 (0.11)	22.50	28.32
25 ± 1	4.42 (0.02)	15.00 (0.13)	16.43	18.08
32 ± 1	2.51 (0.01)	18.00 (0.10)	17.26	16.20
32 ± 1	3.59 (0.04)	12.00 (0.09)	13.65	11.57
32 ± 1	4.42 (0.02)	10.50 (0.04)	11.13	7.64

### Predictive model of TTD of SEA using the threshold method

**Figure 4:**
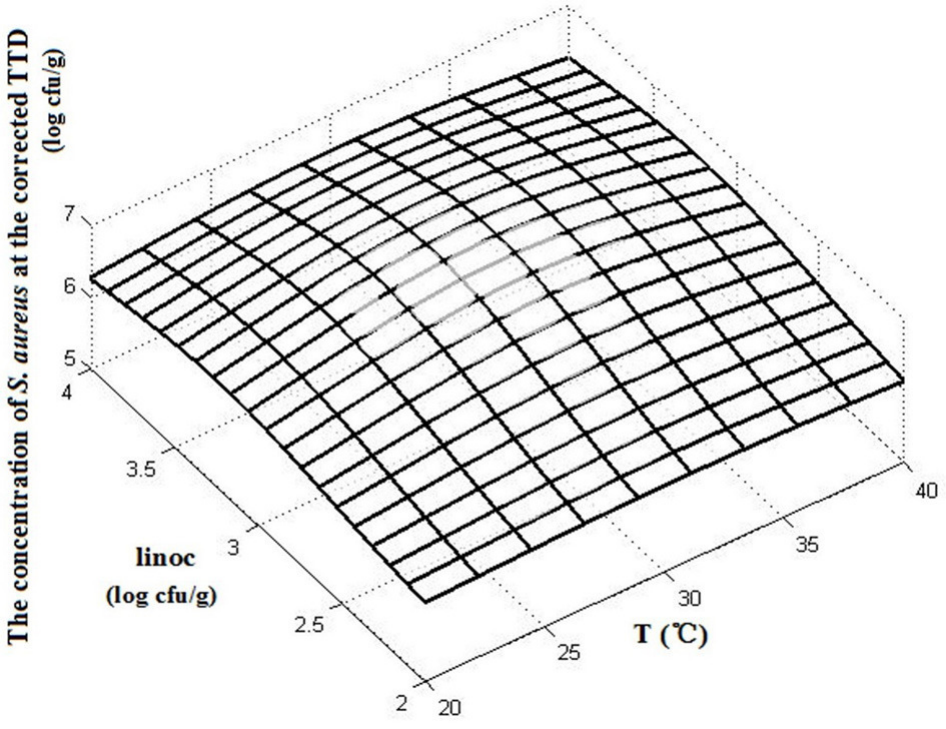
The concentration of *S. aureus* at the corrected TTD under various conditions.

Table 3 shows the concentration of *S. aureus* at the corrected TTD of SEA under designed environmental conditions. Figure 4 shows the effects of temperature and initial inoculation concentrations on the concentrations of *S. aureus* at the corrected TTD of SEA. A flat surface with a slight fluctuation (Figure 4) indicated that the concentration of *S. aureus* at the TTD of SEA was always within a narrow range regardless of the varying environmental conditions. In other words, SEA could be detected only when the concentration of *S. aureus* reached a particular level. According to the results of this experiment, this particular level (threshold) of *S. aureus* for producing a detectable amount of SEA in cooked chicken meat was estimated to be 6.32 ± 0.35 log CFU/g, which was the mean value of *S. aureus* concentration at the corrected TTD under designed environmental conditions.

The threshold concentration of *S. aureus* could be used as a critical reference to determine whether SEA could be detected. Thus, we considered that the time required to reach the threshold could be calculated by an *S. aureus* growth predictive model. In the present study, we thought the TTD of SEA in cooked chicken meat could be obtained by calculating the time required to reach the concentration of 6.32 log CFU/g using an *S. aureus* growth predictive model in cooked chicken. Thus, this method was called the predictive model of TTD of SEA using the threshold method.

Hu et al. (2016) built the predictive model for *S. aureus* growth in cooked chicken meat, inoculating the same *S. aureus* strain that produces SEA (SA14966) into the cooked chicken meat. Modified Gompertz model, modified logistic model, and Baranyi model were all used to fit the growth curve. The modified Gompertz model was considered the optimal primary model to describe the *S. aureus* growth in cooked chicken meat; this conclusion well agreed with the result of other author (Lee et al., 2015). The secondary model was established by using response surface equation. On the basis of the established primary model and secondary model, the time required to reach a certain *S. aureus* concentration could be calculated under a temperature range of 15–36°C and an initial inoculation concentration range of 10^2^-10^4^ CFU/g within 72 h.

The predicted results obtained by this method for model establishment and model validation are shown in Tables 4, 5, respectively.

### Validation and comparison of the two methods

In this study, both the linear polynomial regression and threshold methods were subjected to internal and external validation. The observed and the predicted values from the two methods are shown in Tables 4, 5.

The temperature of 15°C was considered beyond the interpolation ranges of Equation (7). Therefore, in this study, the data of 22, 29, and 37°C in Table 4 and the data of 25, and 32°C in Table 5 were used for model validation. The threshold concentration of *S. aureus* for producing a detectable amount of SEA in cooked chicken was considered to be 6.32 log CFU/g. However, the concentration of *S. aureus* would never reach this concentration within 72 h under certain conditions. As a result, the predicted values could not be calculated by using the established growth model. Thus, this situation in Tables 4, 5 was marked as “nc (no calculated result).”

**Figure 5:**
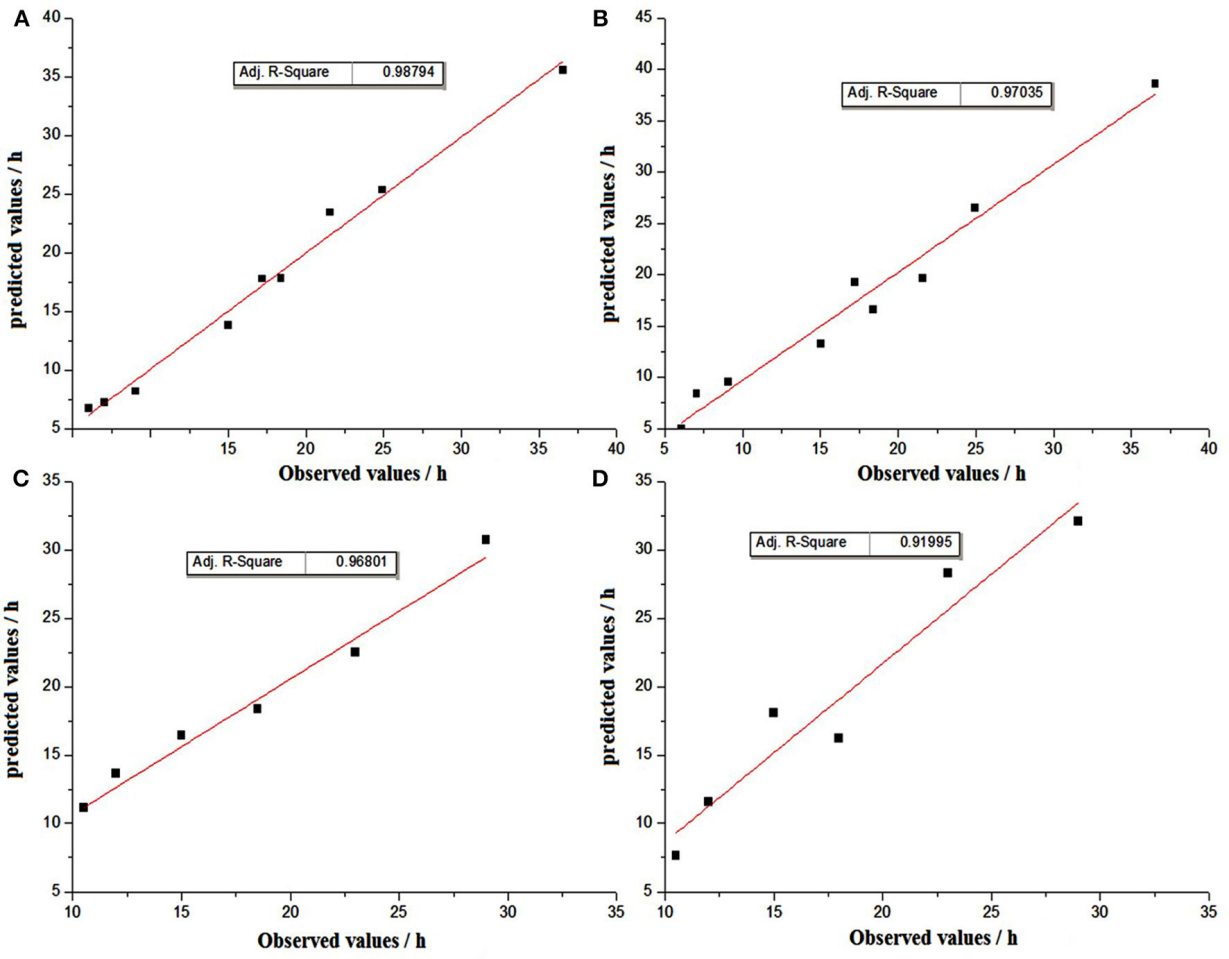
Graphical comparisons beteween observed and predicted TTD values by the method of linear polynomial regression for internal validation **(A)** and external validation **(C)**; by the method of threshold for internal validation **(B)** and external validation **(D)**.

The generalization properties of the models were evaluated by using graphical comparisons, and plots between the observed and predicted TTD of SEA by using linear polynomial regression and the threshold model under experimental conditions are shown in Figure 5.The results of graphical comparisons indicate that the correlation coefficients from internal validation were better than those from external validation. In the internal validation, both methods showed satisfactory regression results, while the prediction of linear polynomial regression method showed a slightly better agreement than the threshold model. In the external validation, the prediction method of linear polynomial regression was also significantly better than the threshold method (Figure 5).

For further evaluation of the established prediction models, *RMSE, B*_*f*_, *A*_*f*_, and *%SEP* were calculated as described by Dong et al. (2007). The results of nine conditions for model establishment (Table 4) were used for internal evaluation, as shown in Table 6. The results of an additional six conditions for model validation (Table 5) were used for external evaluation, as shown in Table 6.

**Table 6:** Mathematical internal and external evaluation based on TTD of SEA by two methods for establishing the model.

**Evaluation validation**	**Method**	***%SEP***	***RSME***	***B*_*f*_**	***A*_*f*_**
Internal validation	Linear polynomial regression	9.4096	1.6288	1.0056	1.1854
	Threshold	22.0439	3.8158	0.9613	1.4839
External validation	Linear polynomial regression	9.6406	1.7353	1.0909	1.1471
	Threshold	24.7093	4.4279	0.4697	2.9622

*RMSE* is used to measure the dispersion of predicted values (Wang et al., 2011). Table 6 shows that the *RMSE* of internal evaluation from the linear polynomial regression method (1.6288) was better than that from the threshold method (3.8158).

*B*_*f*_ is a measure of the extent of under- or over prediction by the model and shows the structural deviation of the prediction model (Dong et al., 2007). Internal evaluation results (Table 6) demonstrated that *B*_*f*_ was close to 1 for both prediction methods (1.0051 and 0.9613). Based on the opinions proposed by Ross (1999), the interpretations of *B*_*f*_ when used for evaluating the model performance involving pathogens was described as following: “0.90–1.05 can be considered good; 0.70–0.90 or 1.06–1.15 can be considered acceptable; and <0.70 or >1.15 should be considered unacceptable.” This standard reflects that the internal evaluation results of *B*_*f*_ are within the good range.

*A*_*f*_ values were also measured in this study. The average estimate tends to be less accurate with the increase in the *A*_*f*_ value, while a value of 1 indicates perfect consistency between all predicted and observed values (Lebert et al., 2000). Ross et al. (2000) proposed that an acceptable accuracy factor could be determined by considering the numbers of environmental variables in a model. Therefore, the best performance that might be expected from the kinetic model that contains the effects of initial inoculation concentration and incubation temperature is an accuracy factor of 1.2. In this study, *A*_*f*_ values of 1.1854 and 1.1471 for the linear polynomial regression method from both internal and external evaluation were within the acceptable range, but the A_*f*_ values of 1.4839 and 2.9622 for the threshold method were unsatisfactory.

Standard error of prediction *(%SEP)* expressed as a percentage has the advantage of being dimensionless (Garcia-Gimeno et al., 2003). A few scientific studies reflect the *%SEP* values. For example, the best values obtained were 14.04% of SEP for the growth rate and 14.84% for the lag estimation of *Lactobacillus plantarum* (Garcia-Gimeno et al., 2002) by the best ANN model, which were much better than those obtained by RSM at 35.63 and 39.30%, respectively. Compared with the results in Tables 6, 7, the *%SEP* values of the linear regression model were much lower than those of the threshold method from both internal and external validation.

Tables 6 shows that the statistical results of RMSE, SEP, and factors *B*_*f*_, *A*_*f*_ of the internal validation were better than those of the external validation, and the linear polynomial regression method was more accurate than the threshold method.

## Discussion and conclusion

A preliminary exploration of the TTD predictive methods was performed in this study. We assumed that the TTD of SEA was a response of *S. aureus* to environmental variables. A mathematical model was used for the first time to quantify the effects of environmental variables on the TTD of SEA in cooked chicken meat. The experiment and validation results indicate the validity of our hypothesis and designed methods, that is to say, the TTD of SEA in food is highly correlated with environmental variables and it can be predicted by establishing a mathematical model that describes the effects of environmental variables on the TTD of SEA.

On the basis of the result of this study, temperature is considered the significant environmental factor that influences the TTD of SEA. The TTD would be delayed effectively when cooked meat products are stored at low temperatures. This condition serves as a reminder for manufacturers that low-temperature storage is critical for extending the shelf life of RTE cooked meat products. According to the local regulation issued by the Shanghai Municipal Food and drug administration (http://www.shfda.gov.cn/gb/node2/yjj/xxgk/zfxxgk/zxxxgk/sp/userobject1ai9285.html), the shelf life of RTE cooked meat products without packaging is within 24 h. On the basis of the calculated results from our established predictive model, if the cooked chicken product was contaminated with SEA-producing *S. aureus* at an initial concentration of approximately 10^3^ CFU/g, then SEA could not be detected within 24 h when the product was stored at a temperature lower than 27.0°C. To ensure safety, the product should be stored at a temperature lower than 22.0°C. Hence, SEA could not be detected within 30.8 h.

Among the mathematical models used in predictive microbiology, linear polynomial regression is a useful method and has been widely used to predict the growth kinetics of bacteria (Zhao et al., 2002; Dong et al., 2007; Lee et al., 2015). The first use of polynomial regression to predict the enterotoxins was reported by Chaves et al. (2017). In that study, temperature, pH value (5.9–6.2) and NaCl concentration (0.8–2.3%) were considered as experimental variables to establish the predictive model, the results of that study confirmed our previous assumption that the pH value and NaCl concentration had no significant effect on TTD. Accordance with our experimental design, temperatures and initial inoculating concentrations were chosen as the environmental variables, the result of correlation test showed that the temperature had a high negative correlation with TTD, which is consistent with the conclusions of Chaves et al. (2017). The initial inoculating concentration also had negative correlation with TTD, but its correlation level was lower than that of temperature. The initial inoculating concentration was chosen as an environmental variable in this study because it can be used to simulate the level of food contamination in practical applications.

Some published papers reported the minimum concentration (threshold) of *S. aureus* for producing a detectable amount of SEA under variable environmental conditions in different foods (Anunciaçao et al., 1995; Fujikawa and Morozumi, 2006; Lin et al., 2015). It was Fujikawa and Morozumi (2006) who first proposed the threshold method in their study on predicting the amount of toxin in milk. The initial time (another expression of TTD) in that study was suggested to be predicted by using the threshold concentration of *S. aureus* and the established *S. aureus* growth model in milk. Tango et al. (2015) also analyzed the *S. aureus* cell density and the time when the toxin could be detected under various temperatures in cooked fish paste. However, both of them didn't apply and validate this method in their article.

To date, the monitoring of enterotoxins production during the incubation was performed by intermittent detection rather than continuous real-time detection, because the latter method could not be applied for practical use (Cretenet et al., 2011). Given the intermittent sampling in studies, the observed TTD of SEA will not be the same as the actual TTD of SEA. Thus, how to determine the actual TTD becomes a problem. To best of our current knowledge, the time of first positive point was usually considered as the earliest time to produce enterotoxins and used as observed value at all studies. In the present study, linear interpolation was used to correct the observed TTD. On the basis of the principle of interpolation calculation (Figure 1), the corrected TTD of SEA is close to the actual TTD. Given the corrected TTD used to the regress the polynomial equation instead of observed TTD, the predicted results obtained from the regressed equation would be more accurate.

Moreover, the use of linear interpolation would be helpful to obtain a more accurate threshold value, which makes the predicted result more accurate. Based on the calculated results of Table 3, the mean value of the *S. aureus* concentration at the observed TTD (7.08 ± 0.48 log CFU/g) was significantly higher (*p* < 0.01) than that at the corrected TTD (6.32 ± 0.35 log CFU/g).Using the former value as the threshold would result an unsafe prediction in practice because the predicted TTD using the former value as the threshold would obviously longer than that using the latter value as the threshold.

The major finding of this study is that both linear polynomial regression and threshold methods are suitable for predicting the TTD of SEA in cooked chicken products despite their different prediction approaches. The use of linear polynomial regression takes advantage of its straightforward approach and absence of knowledge of a particular process, while the threshold method is difficult to construct as it requires a considerable amount of data from microbial counting. However, a recent study reported that the use of a molecular predictive model could save more labor and time to develop more precise models of *S. aureus* (Guan et al., 2017). Also, the threshold method seems closer to the construction of mechanistic models because it contributes important information, i.e., the threshold concentration of *S. aureus* for producing a detectable amount of SEA, which shows great biological significance. Furthermore, the threshold value for *S. aureus* to produce SEA in various foods could be embedded into current microbial modeling software packages to add the function of SEA prediction, thereby significantly increasing the application of microbial modeling software packages.

## Author contributions

JH designed and performed the experiments, analyzed the data and wrote the manuscript. LL prepared reagents, materials and performed the experiments together with JH. MC performed the detection of enterotoxin. WY contributed to the conception of the study, conceived the experiments and approved the final version of the manuscript.

### Conflict of interest statement

The authors declare that the research was conducted in the absence of any commercial or financial relationships that could be construed as a potential conflict of interest.
